# Anophthalmia Plus Syndrome: A Case Report of Severe Ocular and Systemic Anomalies in a Neonate

**DOI:** 10.1155/carm/2780074

**Published:** 2026-09-16

**Authors:** Zainab Abushgair, Khayry Al-Shami, Ziad Haddad, Falah Qudah, Mohammad Al_Maghayreh, Mahdi Alshboul, Yahia Ranjous

**Affiliations:** ^1^ Department of Ophthalmology, Princess Basma Teaching Hospital, Irbid, Jordan; ^2^ Department of Clinical Sciences, Faculty of Medicine, Yarmouk University, Irbid, Jordan, yu.edu.jo; ^3^ Princess Rahma Teaching Hospital, Irbid, Jordan; ^4^ Clinical Science Department, Pediatric Division, Faculty of Medicine, Yarmouk University, Irbid, Jordan, yu.edu.jo; ^5^ Faculty of Medicine, Damascus University, Damascus, Syria, damascusuniversity.edu.sy

**Keywords:** anophthalmia plus syndrome, case report, congenital anomalies, multisystem involvement, ocular malformations

## Abstract

Anophthalmia is a severe congenital ocular malformation characterized by the complete absence of one or both eyes, distinct from microphthalmia, in which the eye is significantly underdeveloped. This case report details a male infant born at 37 weeks of gestation via cesarean section because of a transverse lie and placental abruption, with low birth weight (2100 g), who presented with multiple congenital anomalies including cleft lip and palate, right ear malformation, and severe ocular defects. Despite a prenatal course with limited risk factors and no family history of ocular diseases, postnatal examinations revealed microcornea, microphthalmia, and the absence of the left globe, replaced by cystic tissue. Additional findings included a heart murmur, decreased white matter density on brain ultrasound, and micrognathia. The infant, who developed disseminated intravascular coagulation (DIC) secondary to sepsis, passed away at 21 days of age. This case underscores the complexity of managing anophthalmia, especially when associated with other systemic anomalies, highlighting the need for a multidisciplinary approach, comprehensive genetic evaluation, and supportive care. The unpredictable nature of such anomalies and the limitations of current prenatal care underscore the need for ongoing research and improved diagnostic strategies.

## 1. Introduction

Anophthalmia is a severe congenital ocular malformation characterized by the complete absence of one or both eyes [1]. It differs from microphthalmia, in which the eye is significantly underdeveloped or contains residual neuroectodermal tissue. The term “clinical anophthalmia” describes apparent total absence of the eye; however, microscopic examination often reveals residual ocular tissue. Anophthalmia can manifest as an isolated developmental abnormality or as part of a broader multisystem syndrome [2].

The etiology of anophthalmia is complex and involves genetic and environmental factors. Environmental contributors include certain infections (such as TORCH infections like cytomegalovirus [CMV], rubella, Parvovirus B19, and toxoplasmosis), nutritional deficiencies (particularly vitamin A), and prenatal exposure to toxins (including alcohol, thalidomide, and warfarin). These factors may disrupt early optic development, including neural tube induction, optic vesicle and optic cup formation, or secondary degeneration of ocular tissues [3].

Genetically, anophthalmia is associated with mutations in several genes critical for eye development. Among these, mutations in the SOX2 gene are the most frequently implicated, accounting for up to 40% of anophthalmia cases, and mutations are typically de novo heterozygous loss‐of‐function variants. Anophthalmia caused by SOX2 mutations is usually bilateral and may be part of SOX2 anophthalmia syndrome, which also involves extraocular manifestations such as learning disabilities, growth and motor delays, seizures, and malformations of central nervous system structures [4]. OTX2 mutations, which affect optic vesicle and retinal formation, occur in about 3% of patients with bilateral anophthalmia and may be associated with developmental delay and structural brain abnormalities [5]. Similarly, PAX6, a key regulatory gene in eye development, has been associated with various ocular abnormalities, including anophthalmia, particularly when interacting with SOX2 mutations. PAX6 mutations are also linked to systemic manifestations such as growth delays, seizures, ADHD, and microcephaly [6].

The pathophysiology of anophthalmia involves disruptions of ocular development, which begin as early as the fourth week of gestation. The failure of these processes can result in true anophthalmia, which can be classified into primary, secondary, or consecutive anophthalmia depending on the specific stage of developmental failure or tissue degeneration. True anophthalmia is exceptionally rare, with reported incidences of 0.18–0.33 cases per 10,000 births [7]. Risk factors include older maternal age, multiple births, low birth weight, prematurity, infections, vitamin A deficiency, hyperthermia, and teratogenic substances. Anophthalmia can also occur in the context of genetic syndromes such as trisomy 13, trisomy 18, and mosaic trisomy 9. This complex interplay of genetic and environmental factors underscores the need for further research into its mechanisms, prevention, and management [3]. In this report, we describe a male infant with anophthalmia plus syndrome born at 37 weeks of gestation via cesarean section (C‐section) for transverse lie and placental abruption, with a low birth weight of 2100 g.

## 2. Case Presentation

A male infant was born at 37 weeks of gestation via C‐section, necessitated by a transverse lie and placental abruption, with low birth weight (2100 g). The mother, a 29‐year‐old woman, and the father, aged 31, are both nonsmokers and nonconsanguineous. This was the mother’s third pregnancy, having previously delivered two healthy daughters, aged 6 and 4, through normal vaginal delivery (NVD). Her prenatal course was complicated by a threatened miscarriage, for which she received paracetamol and cefadroxil antibiotics for an upper respiratory tract infection (URTI) during the first trimester. She also received hormonal injections to manage the miscarriage threat. She had no history of radiation exposure.

**FIGURE 1:**
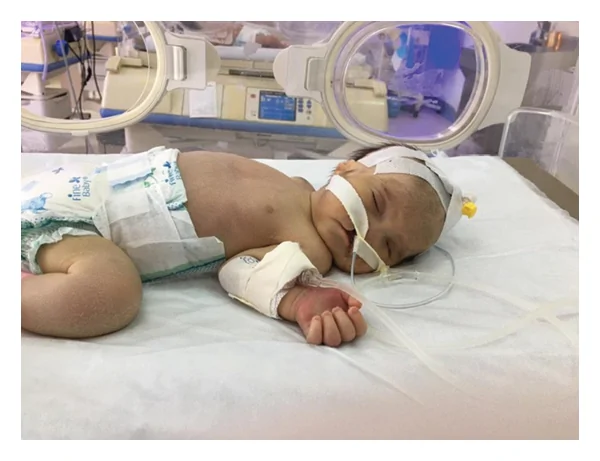
The infant in the neonatal intensive care unit.

**FIGURE 2:**
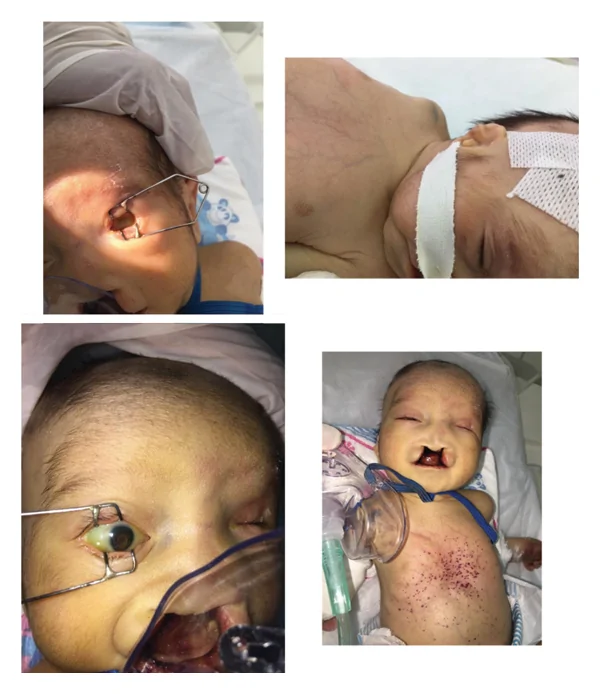
Clinical photographs demonstrating the physical findings and congenital anomalies.

Upon birth, the infant was immediately admitted to the neonatal intensive care unit (NICU) due to multiple congenital anomalies, including a cleft lip and palate, right ear malformation, deformed nostrils, and left eye abnormality (Figure 1). On physical examination, the baby was noted to have low‐set ears with an obliterated right external ear, a depressed nasal bridge, underdeveloped nostrils, a heart murmur, and abnormal‐appearing eyes, particularly on the left side. The neonate also exhibited signs of spasticity (Figure 2).

**FIGURE 3:**
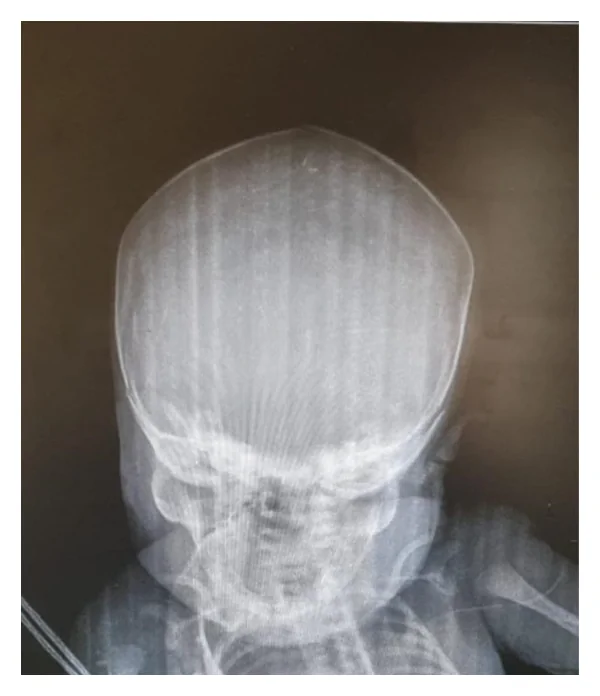
Skull X‐ray shows micrognathia and microcephalus.

Given the complexity of the infant’s condition, consultations with cardiology; ear, nose, and throat (ENT); and ophthalmology were promptly requested. A series of investigations was initiated, including complete blood count (CBC), kidney function tests (KFTs), liver function tests (LFTs), and electrolyte levels. In addition, renal and brain ultrasounds, an echocardiogram (ECHO), and a skull X‐ray were ordered (Figure 3).

In the first 2 weeks of life, the laboratory results were generally unremarkable, except for low calcium levels. However, the baby subsequently developed hyponatremia and hyperkalemia, raising concerns about a possible endocrine disorder. The ECHO revealed an atrial septal defect (ASD) primum, while the renal ultrasound was unremarkable. The brain ultrasound showed decreased density of the white matter without midline shifting, hydrocephalus, or hemorrhages. The skull X‐ray revealed micrognathia and microcephalus. Although the orbital sizes appeared equal, the left eye was noted to be smaller, likely due to its position; unfortunately, further imaging with CT or MRI was not pursued at that time due to resource constraints.

The ophthalmology examination provided more insights: the right eye exhibited microcornea, possible microphthalmia, mild corneal haze, inferior iris coloboma, and chorioretinal coloboma involving the optic nerve. No gross cataract was observed, although intraocular pressure (IOP) was not checked. The left eye was more severely affected, with an underdeveloped eyelid and complete absence of the globe, replaced with cyst‐like tissue. Unfortunately, despite the best efforts to manage his complex condition, the baby developed disseminated intravascular coagulation (DIC) secondary to sepsis. Tragically, he passed away at the age of 21 days.

## 3. Discussion

The case of this male infant, born at 37 weeks of gestation via C‐section, illustrates the challenges associated with managing congenital anomalies, particularly those related to anophthalmia and associated syndromes. The presence of multiple congenital anomalies, including anophthalmia, cleft lip and palate, right ear malformation, and other craniofacial abnormalities, underscores the importance of a multidisciplinary approach in such cases. Early involvement of specialties such as cardiology, ENT, and ophthalmology was crucial in assessing and attempting to manage the infant’s condition [8].

Anophthalmia, as seen in this case, is a rare but severe congenital anomaly characterized by the complete absence of the globe. The complexity of this condition is compounded by its association with other anomalies, as evident in this infant, where additional craniofacial and systemic anomalies were present. The difficulty in prenatal detection of anophthalmia, particularly when no other major congenital anomalies are identified, underscores the challenges in early diagnosis and preparation for the necessary postnatal care [4]. The prenatal course of the mother, which included complications such as a threatened miscarriage and the use of medications such as paracetamol and antibiotics, did not provide clear predictive factors for the severity of the congenital anomalies observed in the infant. This highlights the unpredictable nature of such anomalies and the potential limitations of prenatal care in identifying and managing these risks. The absence of a family history of ocular diseases and the lack of consanguinity also add to the complexity of understanding the etiology of this case.

The findings from the ophthalmology examination, which revealed significant abnormalities such as microcornea, microphthalmia, coloboma, and the absence of the left globe, are indicative of the severe nature of the ocular defects. These findings are consistent with the spectrum of conditions associated with anophthalmia and microphthalmia, which often include other systemic anomalies, as was evident in the heart murmur and abnormal brain ultrasound findings in this case. The lack of further imaging, such as CT or MRI, represents a limitation in fully characterizing the extent of the craniofacial and ocular abnormalities [9]. Anophthalmia plus syndrome, which was considered in this case, is a rare condition that involves severe ocular malformations along with other systemic anomalies. The association of anophthalmia with septo‐optic dysplasias, as well as other craniofacial abnormalities, further complicates the clinical management and prognosis of affected individuals [10]. The genetic underpinnings of such syndromes are often complex and may involve multiple genes, as suggested by recent studies. However, the exact genetic cause in this case remains undetermined, highlighting the need for further genetic evaluation and research in similar cases. Table 1 summarizes previously reported cases with similar clinical features.

**TABLE 1:** Illustration for similar cases of Microphthalmia–anophthalmia plus syndrome.

Case	Patient details	Clinical findings	Imaging findings	Diagnosis	Additional notes
1	Term male infant, healthy nonconsanguineous parents [11]	Bilateral cleft palate and lip, mild microphthalmia, iris coloboma, glaucoma, blepharophimosis, spina bifida, caudal tethering of spinal cord, filum terminalis lipoma, syringomyelia, hypoplasia of corpus callosum	Spine radiograph and MRI: first sacral hemivertebra, agenesis of sacral vertebrae and coccyx, caudal tethering, lipoma, syringomyelia; Brain ultrasound and MRI: hypoplasia of corpus callosum, mild dilatation of lateral ventricles; Orbital MRI: bilateral microphthalmia, distorted left eyeball, split vitreous body	Anophthalmia‐plus syndrome (APS)	Gene locus of APS yet to be identified
2	1‐week‐old female neonate, born at 39 weeks [12]	Persistently closed eyelids, inability to palpate globes	Sonography: empty and hypoplastic orbital cavities, bilateral absence of globes	Congenital bilateral anophthalmia	Emphasizes reliance on ultrasonography in Sub‐Saharan Africa
3	1‐year‐old male, born via caesarean delivery [13]	Absence of eyeballs, bluish mass in lower eyelids, history of unilateral cleft lip and palate	Imaging: congenital cystic eyeballs, bilateral microphthalmia, enlarged orbits, maxillary sinus hypoplasia, mild ventriculomegaly	Cyst removal, orbital reconstruction needed	Follow‐up required
4	3‐year‐old male with APS [14]	Asymmetry of face and head, left choanal atresia, microphthalmia, bilateral low‐set ears, cleft palate, 45 dB conductive hearing loss	MRI: decreased right globe volume, undeveloped left globe, decreased left optical nerve thickness, Chiari Type 2 malformation, left choanal atresia, cleft palate; echocardiography and abdominal ultrasonography: normal	Fryns anophthalmia plus syndrome	Includes central hypothyroidism, developmental regression
5	Newborn male, delivered vaginally, birth weight 2.7 kg [15]	Absent right eye, left cleft lip and palate	Imaging: small right orbital cavity, absent right eye, hypoplastic right cranium, absent cranial bones, large porencephalic cysts communicating with right lateral ventricle, dilated occipital horns	Cranial abnormalities with cleft lip and palate	Mother had no history of diabetes, hypertension, drug exposure, or consanguinity

Despite the multidisciplinary efforts to manage the infant’s condition, the development of DIC secondary to sepsis ultimately led to the infant’s demise at 21 days of life. This outcome emphasizes the severe prognosis associated with complex congenital anomalies, particularly when multiple systems are involved. The challenges in managing such cases extend beyond the immediate postnatal period, with long‐term considerations including the potential for further complications and the need for ongoing supportive care [4].

The main limitations of this case include the absence of CT or MRI to definitively exclude intraorbital remnants of the left globe. Furthermore, genetic testing was not performed because the results would not have altered either clinical management or the overall prognosis. Nevertheless, the absence of genetic testing prevents the definitive exclusion of overlapping or concurrent syndromic conditions.

This case underscores the importance of early and comprehensive evaluation of infants born with multiple congenital anomalies. The complexity of conditions such as anophthalmia, especially when associated with other systemic anomalies, requires a coordinated approach involving multiple specialties. Advances in genetic research may eventually provide more insights into the etiology and management of these challenging cases, but in the meantime, a focus on supportive care and the prevention of complications remains paramount.

## Funding

The authors did not receive funding for this paper.

## Ethics Statement

This study is exempt from ethical approval in our institution.

## Consent

Written informed consent was obtained from the patient’s parents for publication of this case report and accompanying images. A copy of the written consent is available for review by the Editor‐in‐Chief of this journal on request.

## Conflicts of Interest

The authors declare no conflicts of interest.

## Data Availability

The data that support the findings of this study are available from the corresponding author upon reasonable request.

## References

[1] FitzPatrick D. R. and van Heyningen V. , Developmental Eye Disorders, Current Opinion in Genetics & Development. (2005) 15, no. 3, 348–353, 10.1016/j.gde.2005.04.013. 15917212

[2] Plaisancié J. , Ceroni F. , Holt R. et al., Genetics of Anophthalmia and Microphthalmia. Part 1: Non-Syndromic Anophthalmia/Microphthalmia, Human Genetics. (2019) 138, no. 8-9, 799–830, 10.1007/s00439-019-01977-y. 30762128

[3] Verma A. S. and Fitz Patrick D. R. , Anophthalmia and Microphthalmia, Orphanet Journal of Rare Diseases. (2007) 2, 1–8, 10.1186/1750-1172-2-47. 18039390 PMC2246098

[4] Williamson K. A. and Fitz Patrick D. R. , The Genetic Architecture of Microphthalmia, Anophthalmia and Coloboma, European Journal of Medical Genetics. (2014) 57, no. 8, 369–380, 10.1016/j.ejmg.2014.05.002. 24859618

[5] Mauri L. , Franzoni A. , Scarcello M. et al., SOX2, OTX2 and PAX6 Analysis in Subjects With Anophthalmia and Microphthalmia, European Journal of Medical Genetics. (2015) 58, no. 2, 66–70, 10.1016/j.ejmg.2014.12.005. 25542770

[6] Fuhrmann S. , Eye Morphogenesis and Patterning of the Optic Vesicle, Current Topics in Developmental Biology. (2010) 93, 61–84. 20959163 10.1016/B978-0-12-385044-7.00003-5 PMC2958684

[7] Sadler T. W. , Langman’s Medical Embryology, 2022, Lippincott Williams & Wilkins.

[8] Chuka-Okosa C. , Magulike N. , and Onyekonwu G. , Congenital Eye Anomalies in Enugu, South-Eastern Nigeria, West African Journal of Medicine. (2005) 24, no. 2, 112–114, 10.4314/wajm.v24i2.28178. 16092309

[9] Jana M. and Sharma S. , Bilateral Anophthalmia With Septo-Optic Dysplasia, Oman Journal of Ophthalmology. (2010) 3, no. 2, 86–88, 10.4103/0974-620x.64233. 21217902 PMC3003857

[10] Katowitz W. R. , Anophthalmia and Microphthalmia, Atlas of Orbital Imaging, 2021, Springer, 209–213.

[11] Makhoul I. R. , Soudack M. , Kochavi O. , Guilburd J. N. , Maimon S. , and Gershoni‐Baruch R. , Anophthalmia‐Plus Syndrome: A Clinical Report and Review of the Literature, American Journal of Medical Genetics, Part A. (2007) 143, no. 1, 64–68, 10.1002/ajmg.a.31566. 17152069

[12] Izevbekhai O. S. , Eluehike S. U. , and Irabor P. F. I. , A Rare Case of Congenital Bilateral Anophthalmia in Irrua: A Case Report, Journal of Medical Case Reports and Reviews. (2023) 6, no. 05, 1274–1278.

[13] Kumar N. , Tenagi A. L. , and Powar R. , Bilateral Anophthalmia Plus Syndrome: A Case Report, Delhi Journal of Ophthalmology. (2019) 29, no. 4, 77–79, 10.7869/djo.450.

[14] Cayir A. , Tasdemir S. , Eroz R. , Yuce I. , Orbak Z. , and Tatar A. , Anophthalmia-Plus Syndrome With Unusual Findings. A Clinical Report and Review of the Literature, Genetic Counseling. (2013) 24, no. 3, 307–312. 24341146

[15] Al-Awaysheh F. , Sunna N. , and Besharat A. , Anophthalmia Cleft Lip and Palate Plus Syndrome: Case Report, Journal of Royal Medical Services. (2016) 102, no. 3019, 1–4, 10.12816/0023362.

